# Single-Crystal Elasticity of α-Hydroquinone—An Analogue for Organic Planetary Materials

**DOI:** 10.1021/acsearthspacechem.4c00322

**Published:** 2025-01-01

**Authors:** Jin S. Zhang, Wen-Yi Zhou, Tuan H. Vu, Robert Hodyss, Xinting Yu

**Affiliations:** † Department of Geology and Geophysics, Texas A&M University, College Station, Texas 77845, United States; ‡ Jet Propulsion Laboratory, California Institute of Technology, Pasadena, California 91109, United States; § Department of Physics and Astronomy, University of Texas at San Antonio, San Antonio, Texas 78249, United States

**Keywords:** α-hydroquinone, elasticity, Titan, Brillouin spectroscopy, Dragonfly

## Abstract

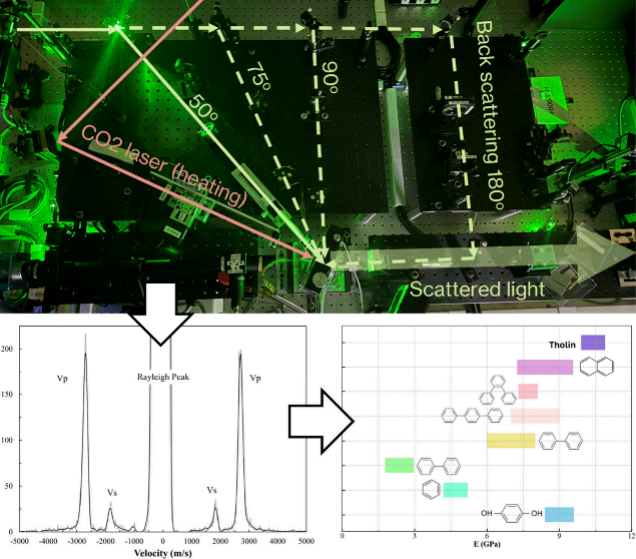

In this study, we measured the single-crystal elasticity of α-hydroquinone at ambient conditions using Brillouin spectroscopy to assess the feasibility of this technique for studying the mechanical properties of organic ices in the outer solar system. In this study, α-hydroquinone serves as an ambient temperature analogue for low-temperature organic ices on Titan and other solar system bodies. We found that a satisfactory Brillouin spectrum can be obtained in less than 5 min of experimental time with negligible damage to the sample. The best fit single-crystal elastic moduli of α-hydroquinone were determined as *C*_11_ = 13.67(8) GPa, *C*_33_ = 10.08(6) GPa, *C*_44_ = 4.54(5) GPa, *C*_12_ = 6.9(7) GPa, *C*_13_ = 7.02(7) GPa, *C*_14_ = 0.54(4) GPa, *C*_25_ = 0.51(9) GPa, and *C*_66_ = (*C*_11_ – *C*_12_)/2 = 3.4(3) GPa, with bulk modulus *K*_S_ = 8.7(2) GPa and shear modulus *G* = 3.4(3) GPa. These results demonstrate that Brillouin spectroscopy is a powerful tool for characterizing the elastic properties of organic materials. The elastic properties of organic ices can be broadly applied to understand planetary surface processes and also aid in evaluating the feasibility and technical readiness of future lander, sampling, and rover missions in the outer solar system.

## Introduction

Titan, Saturn’s largest moon, is the only body in the solar system aside from Earth with a substantial atmosphere (95–99% N_2_ along with 1–5% CH_4_) and standing liquids on its surface. Photochemical dissociation and ionization of methane in the presence of N_2_ by solar photons, magnetospheric electrons, and galactic cosmic rays culminate in a broad array of organic molecules ranging from simple (e.g., C_2_H_6_, C_2_H_2_, C_6_H_6_, and HCN) to complex, of which many have been detected in appreciable abundances.^1−3^ The simple organics are produced in the gas phase in the upper atmosphere. However, almost all of them would be cold-trapped in the stratosphere, forming ice clouds,^4^ and most of the simple organics would land on the surface remaining in the solid phase, including exotic ices, such as C_2_H_2_, C_6_H_6_, HCN, C_3_H_4_ (allene and propyne), acetonitrile (CH_3_CN), cyanogen (C_2_N_2_), etc.^5,6^ In fact, only a few simple organic species would land on Titan’s surface as liquids, including CH_4_, C_2_H_6_, C_3_H_8_, and C_3_H_6_, or not condense at all (C_2_H_4_). These simple organics, when they are in the upper atmosphere, could further polymerize and coagulate to form complex refractory organics that are believed to comprise most of Titan’s thick haze layer (the laboratory analogue is the so-called “tholins”^7^) and would also land on Titan’s surface as solids.^8,9^ However, many fundamental properties of solid organic materials (including simple organic ices and complex organic solids) on Titan remain largely unexplored and undetermined. NASA’s upcoming Dragonfly mission to Titan, planned for arrival in the mid-2030s, will perform *in situ* chemical measurements of Titan’s surface materials.^10^ In preparation for the mission, an important piece of information that will help maximize its scientific return is knowledge regarding the mechanical properties (e.g., bulk and elastic moduli) of candidate surface materials.^11^

The sole direct mechanical property measurement on Titan’s surface is a single-point penetrometer experiment done by the Huygens lander, revealing complex layering of Titan’s surface materials.^12^ These materials are likely a mixture of simple and complex organic solids, alongside the water ice bedrock and potentially ammonia ice generated from cryovolcanism.^13^ However, the dearth of available data on the mechanical properties of candidate surface materials has limited our ability to fully interpret the Huygens data. To date, there exists a single mechanical property measurement of one tholin sample produced by the Planetary Haze Research facility, PHAZER, at Johns Hopkins University using a single gas mixture.^14^ For simple organics, the deformation characteristics of benzene, a putative Titan surface material, have only begun to be explored under cryogenic temperatures.^15^ As such, many additional mechanical property measurements need to be performed to yield comprehensive data sets that can improve our understanding of Titan’s dynamic surface and geology.

In addition to Titan, organic ices exist in the atmospheres/on the surfaces of other solar system bodies^16^ that are of future exploration interest, such as Neptune’s moon Triton,^17^ Pluto and its moon Charon,^18^ and many Kuiper belt objects.^19^ Understanding the fundamental mechanical properties of these materials would allow us to answer many scientific questions that are relevant to surface processes in our solar system’s planetary bodies^14,20−23^ and planet formation.^24^

One of the most valuable tools for characterizing mechanical properties at a fundamental level is Brillouin spectroscopy.^25^ This scattering technique, which measures the frequency shift from the interaction of light with acoustic phonons (lattice vibrations) within a material, allows for direct sound velocity measurements of optically transparent materials.^25,26^ With known density values, this technique allows for the precise estimation of single-crystal and aggregate elastic moduli, often with less than 1% uncertainty.^25^ Over the past few decades, Brillouin spectroscopy has been widely used to study the mechanical properties of various materials, such as novel superhard materials,^27,28^ novel nanocrystalline material,^29^ biomaterials,^30,31^ and, most importantly, silicate minerals under high-pressure–temperature conditions for understanding the seismic observations within the Earth’s interior.^32−34^ However, Brillouin spectroscopy has not yet found widespread use in planetary science, particularly for investigating the mechanical properties of planetary surface materials in preparation for upcoming and future ice body missions. In this study, we explore its utility in studying Titan’s surface analogues by conducting single-crystal elasticity measurements of hydroquinone, a derivative of benzene with two hydroxyl groups in the *para* position. The single-crystal elastic properties of hydroquinone have not been experimentally measured before. Aside from being a possible proxy for Titan surface materials (as an example of an organic molecular solid under ambient conditions), hydroquinone is selected due to its ease of forming large clear euhedral single crystals, which are well-ordered and homogeneous, ideal for Brillouin spectroscopy measurements.

## Experimental Methods

ReagentPlus-grade (>99%) hydroquinone was procured from Sigma-Aldrich. A clear euhedral single crystal approximately 80 × 20 × 250 μm in size was hand-selected and inspected under a microscope to ensure that it was free of inclusions and twinning. The crystal was mounted on a three-circle goniometer head and measured by the Rigaku XtaLAB Synergy diffractometer with Ag source and Dectris CdTe detector at the X-ray diffraction (XRD) laboratory in the Department of Chemistry at Texas A&M University (TAMU). The ϕ angle range for the XRD experiments was ±180° with 0.5° step and 5 s exposure time per frame. A total of 1916 reflections (426 unique reflections) were collected and indexed. Unit cell parameters and orientation matrix were subsequently determined using the software CrysAlis Pro. The crystal was confirmed to be α-hydroquinone with a space group of *R*3̅,^35^ and the unit cell parameters were constrained as *a*_1_ = *a*_2_ = *a*_3_ = 38.556(5) Å, *c* = 5.6660(6) Å, α = β = 90°, γ = 120°, and *V*_0_ = 7294(1) Å^3^, with a calculated density (ρ) of 1.3644(2) g/cm^3^. The Miller indices of the largest exposed surface were determined as (2, −1, −1, 0). The orthogonal Cartesian coordinates used for single-crystal elasticity followed the convention of *X*//*a*_1_, *Z*//*c*, and *Y* intersecting with *a*_2_ at an angle of 30° but perpendicular to both *a*_1_ and *c*.

**Figure 1:**
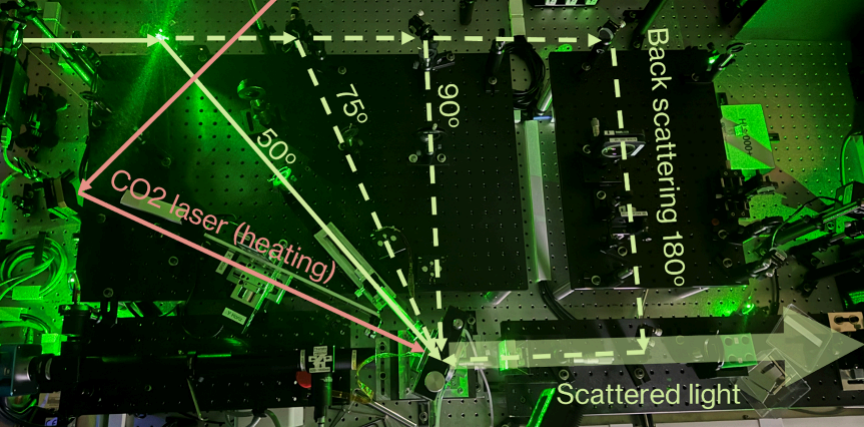
Brillouin spectroscopy system layout in the Geospectroscopy Laboratory at TAMU. Green solid and dashed lines indicate the 532nm green laser paths. Salmon color solid lines indicate CO_2_ laser path for laser-heating the sample (not used in this study). The scattered light is analyzed by TFP1, which was not shown in the figure.

**Figure 2:**
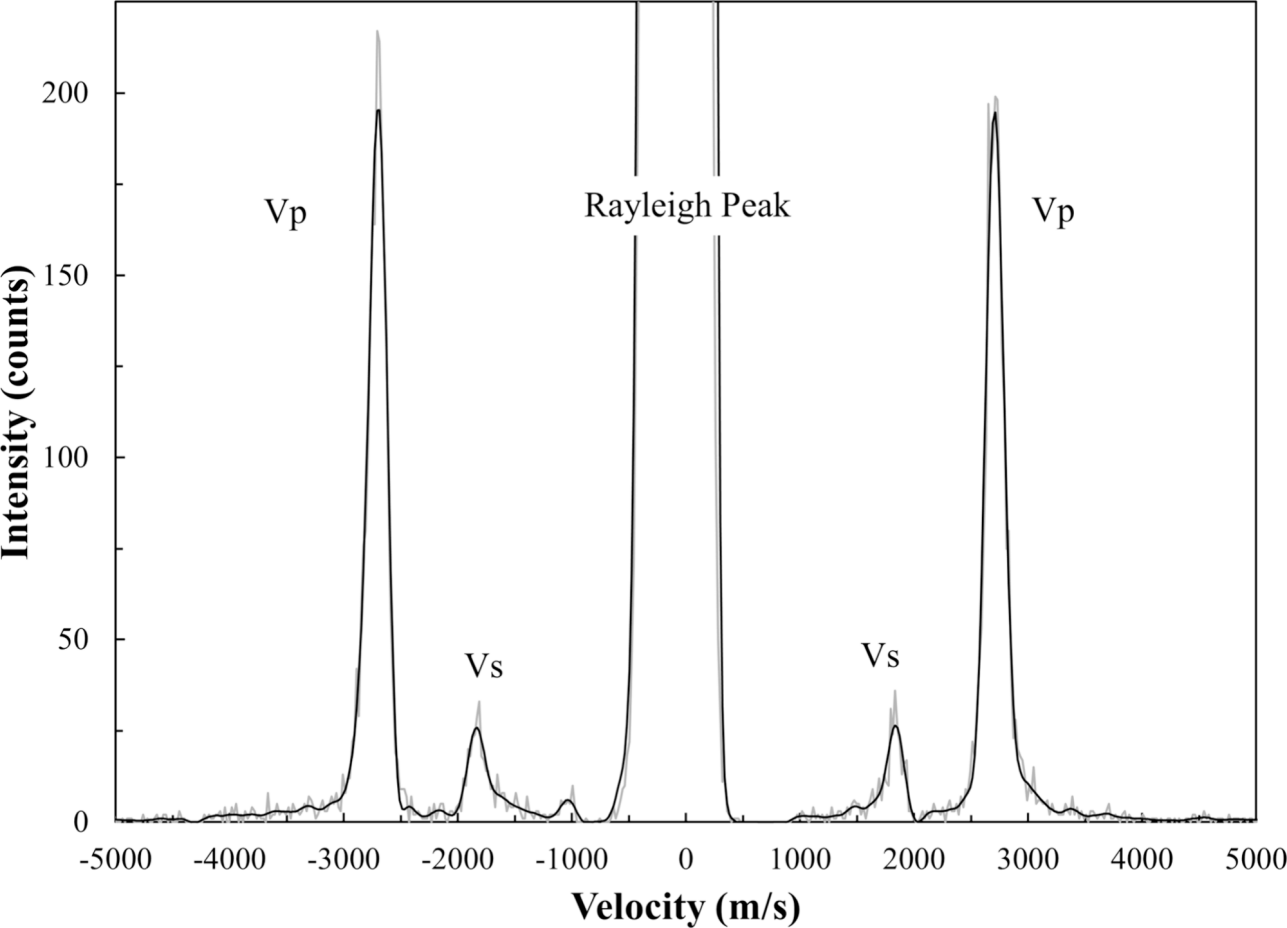
Representative Brillouin spectrum of the α-hydroquinone sample at χ = 6°. The collection time was 5 min.

Brillouin spectroscopy measurements were performed on the selected α-hydroquinone single crystal in the Geospectroscopy Laboratory at TAMU. A 300 mW single-mode diode-pumped solid-state laser with a wavelength (λ_0_) of 532 nm was used as the light source. Brillouin frequency shifts were resolved using a six-pass tandem Fabry–Pérot interferometer TFP1. The detailed experimental setup of the Brillouin spectroscopy system has been described elsewhere,^36^ and the system layout at TAMU was shown in Figure 1. All experiments were conducted using a 50° symmetric forward scattering geometry. The scattering angle (θ) was calibrated to be 50.18(2)° before the experiments using a standard Corning 7980 silica glass, whose velocities have been precisely calibrated using gigahertz ultrasound interferometry.^27^ The sample crystal was placed on top of the culet of a diamond anvil cell for the Brillouin spectroscopy experiments. The laser power was reduced to approximately 15 mW using a wire grid polarizer to minimize laser damage. The incident laser beam entered the largest crystal face (2, −1, −1, 0) from one side and exited the (−2, 1, 1, 0) face on the other side. Under cross-polarized illumination with a petrographic microscope, a uniform interference color was observed, suggesting even thickness across the crystal. The free spectral range was calibrated before and after each experimental run under the reflection mode of the interferometer, and the obtained nonlinearity is within 0.04%. The typical velocity measurement uncertainty was less than 0.03 km/s. The sample was oriented on the system in a way that the phonon wave vector was close to [001] when χ = 0°. As shown in Table 1, longitudinal (*V*_p_) and shear (*V*_s_) wave velocities were measured at a total of 15 different χ angles (6°, 13°, 28°, 43°, 58°, 73°, 88°, 96°, 103°, 118°, 133°, 148°, 163°, 178°, and 180°). Each spectrum was collected for approximately 5 min, yielding spectra with excellent signal-to-noise ratios. A typical Brillouin spectrum is shown in Figure 2. After the experiment, the sample surface was examined under a microscope and confirmed to be free of damage.

**Table 1:** Acoustic Velocities of Single-Crystal α-Hydroquinone along Different Phonon Directions *n* Defined under Orthogonal Cartesian Coordinates^a^

χ (deg)	*n*			*V*_p_ (m/s)	*V*_s_ (m/s)
6	0	0.1045	0.9945	2749.5	1768.5
13	0	0.225	0.9744	2814	1643.5
28	0	0.4695	0.8829	3034.5	1409
43	0	0.682	0.7314	3152.5	1301.5
58	0	0.848	0.5299	3187.5	1503
73	0	0.9563	0.2924	3142	1779.5
88	0	0.9994	0.0349	3124.5	1856
96	0	0.9945	–0.1045	3196.5	1805.5
103	0	0.9744	–0.225	3233.5	1747
118	0	0.8829	–0.4695	3347	1437.5
133	0	0.7314	–0.682	3310	1292
148	0	0.5299	–0.848	3140	1387.5
163	0	0.2924	–0.9563	2873	1613.5
178	0	0.0349	–0.9994	2716	1831
180	0	0.0001	–0.9999	2712	1834

a Only one *V*_s_ has been observed.

## Data Analysis Procedure

Under a symmetric forward scattering geometry, the Brillouin frequency shift Δ*f* is related to the velocity (*V*) along the probed phonon direction, the laser wavelength (λ_0_), and the experimental scattering angle (θ) between the incident and scattered light following the equation below.^26,36,37^
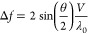
1Thus, acoustic velocities can be calculated from the Δ*f* values obtained from the Brillouin spectra using eq 1. By combining the crystal orientation derived from single-crystal XRD and the acoustic velocities measured from Brillouin spectroscopy experiments along different crystallographic directions, we determined the single-crystal elastic moduli of α-hydroquinone. This was achieved through a least-squares inversion of the Christoffel equation
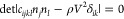
2where *c*_*ijkl*_ is the fourth-order elasticity tensor, *n*_*j*_ and *n*_*l*_ are the directional cosines of the phonon wave vector, ρ is the density, and δ_*ik*_ is the Kronecker delta.^38^

The inversion result based on eq 2 is independent of the assumed initial elasticity tensor model. Due to the relatively high symmetry of trigonal α-hydroquinone, accurate sound velocity measurements within the (2, −1, −1, 0) plane that is parallel to the crystallographic *c* axis allow sufficient constraints to determine its full elasticity tensor.

## Results and Discussion

**Figure 3:**
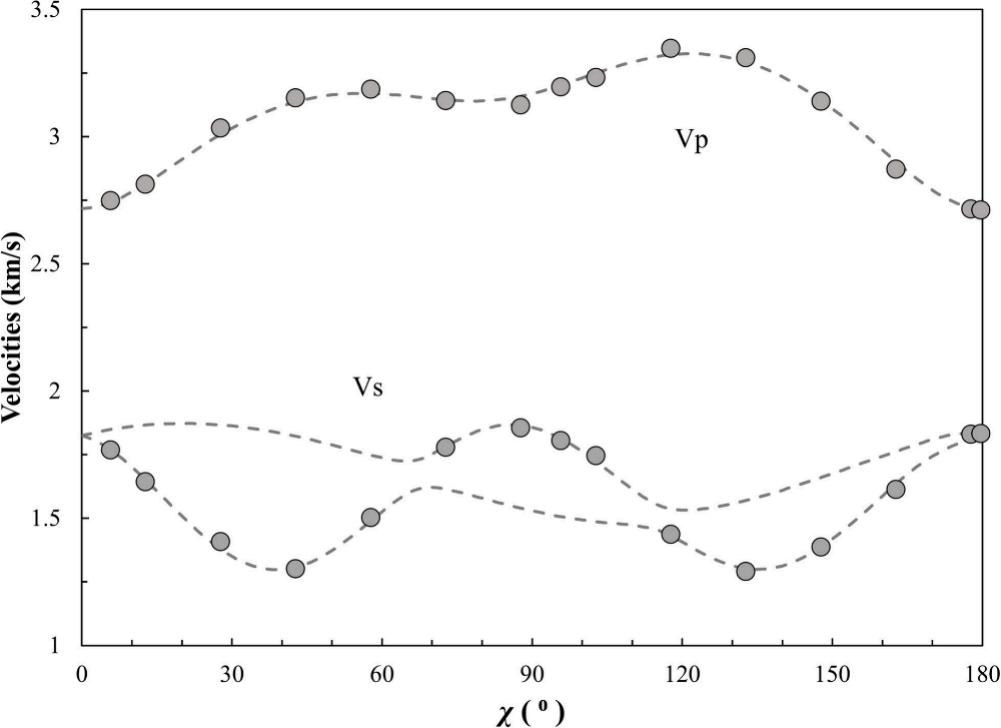
Acoustic velocities as a function of the laboratory angle. The dashed curves are calculated from the best fit single-crystal *C*_*ij*_ model. Gray circles are the actual measurements, whose uncertainties are smaller than the symbols.

α-Hydroquinone has a space group of *R*3̅, resulting in seven independent single-crystal elastic moduli. As shown in Table 2, the best fit single-crystal elastic moduli are *C*_11_ = 13.67(8) GPa, *C*_33_ = 10.08(6) GPa, *C*_44_ = 4.54(5) GPa, *C*_12_ = 6.9(7) GPa, *C*_13_ = 7.02(7) GPa, *C*_14_ = 0.54(4) GPa, and *C*_25_ = 0.51(9) GPa. For a trigonal crystal, *C*_66_ equals (*C*_11_ – *C*_12_)/2, which is estimated to be 3.4(3) GPa for α-hydroquinone. The final inversion root mean square (RMS) error is only 0.015 km/s, indicating an excellent match between the measured acoustic velocities of the crystal (Table 1) and the velocities predicted by the best fit *C*_*ij*_ model (Figure 3). The Voigt–Reuss–Hill (VRH) averaging scheme^39^ was employed to calculate the averaged bulk modulus (*K*_S_) and shear modulus (*G*) as well as the aggregate *V*_p_ and *V*_s_. The Reuss and Voigt bounds as well as the Hill average are presented in Table 2. Young’s modulus *E* and Poisson’s ratio ν are then calculated from *K*_S_ and *G* as 9.0(6) GPa and 0.33(1), respectively. In addition, we calculated the universal anisotropy index (*A*^U^) of α-hydroquinone to be 0.6(2) using the following equation:^40^
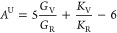
3where the subscripts V and R denote the Voigt and Reuss bounds, respectively. *A*^U^ quantifies the overall elastic anisotropy for materials with arbitrary symmetry.

**Table 2:** Single-Crystal and Aggregate Elastic Properties of α-Hydroquinone^a^

	value	uncertainty		value	uncertainty
*C*_11_ (GPa)	13.67	0.08	ρ (g/cm^3^)	1.3644	0.0002
*C*_33_ (GPa)	10.08	0.06	*K*_V_ (GPa)	8.81	0.15
*C*_44_ (GPa)	4.54	0.05	*G*_V_ (GPa)	3.59	0.08
*C*_12_ (GPa)	6.9	0.7	*K*_R_ (GPa)	8.60	0.15
*C*_13_ (GPa)	7.02	0.07	*G*_R_ (GPa)	3.22	0.08
*C*_14_ (GPa)	0.54	0.04	*K*_S_ (GPa)	8.70	0.21
*C*_25_ (GPa)	0.51	0.09	*G* (GPa)	3.41	0.25
*C*_66_^b^ (GPa)	3.4	0.3	*V*_p_ (km/s)	3.12	0.04
			*V*_s_ (km/s)	1.58	0.05

a Subscripts V and R denote the Voigt and Reuss bounds. *K*_S_ and *G* are calculated as the Voigt–Reuss–Hill average.

b *C*_66_ is calculated as (*C*_11_ – *C*_12_)/2.

**Figure 4:**
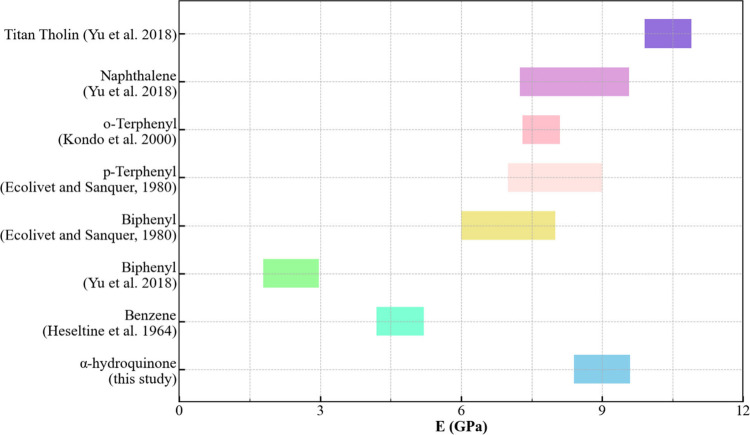
Young’s modulus of various PAHs and Titan tholin compared to α-hydroquinone.

In comparison to benzene and its derivatives, the bulk, shear, and Young’s moduli of α-hydroquinone are similar to those of deuterated biphenyl, *p*-terphenyl, and naphthalene,^14,41^ while benzene and *o*-terphenyl exhibit significantly lower elastic moduli (Table 3 and Figure 4).^42,43^ The elastic moduli of non-deuterated biphenyl obtained from nanoindentation measurements^14^ are substantially lower than those of deuterated biphenyl determined by Brillouin spectroscopy.^41^ While substitution of hydrogen with deuterium might lead to noticeable changes in elastic moduli, the significantly lower Young’s modulus derived from nanoindentation experiments is unlikely to be explained solely by isotopic substitution. It is worth noting that the Young’s modulus of benzene determined by nanoindentation is also significantly lower what was estimated from sound velocity measurements.^15^ This inconsistency is likely due to variations in the samples used across different studies, particularly regarding grain size and fabrication methods,^15^ with potential contributions from the lattice preferred orientations of these strongly anisotropic organic crystals, as indicated by the high *A*^U^ values presented in Table 3. If the aggregate sample tested in the nanoindentation measurements exhibits any preferred orientations of individual crystals, deviations from the ideal averaged elastic moduli based on the VRH scheme would be expected. In addition, the nanoindentation measurements acquired an elastic modulus measured at the surface level of the sample, which could be affected by surface roughness and tip geometry, while sound velocity measurements reflect bulk properties of the ice. The *K*_S_ and *G* values of non-deuterated biphenyl reported in Table 3 are calculated on the basis of the Young’s modulus measured by nanoindentation^14^ under the assumption that the biphenyl sample was a randomly oriented homogeneous and isotropic polycrystalline aggregate, which might not be realistic. A simple polyphenyl chain model has been proposed to explain the systematics observed for the elastic moduli of biphenyl and *p*-terphenyl, particularly for those that are closely related to the longitudinal waves propagating along the molecular long axis.^41^ The fact that α-hydroquinone exhibits elastic moduli similar to those of *p*-terphenyl suggests that directional, relatively strong hydrogen bonding enhances its structural rigidity.^46^ The polycyclic aromatic hydrocarbons (PAHs) listed in Table 3 and Figure 3 have been used as analogues for studying the mechanical properties of Tian’s surface.^14^ In comparison to tholin, all PAHs are notably weaker, as is α-hydroquinone measured in this study. To date, all of the organic candidate materials on Titan’s surface appear to be mechanically much weaker compared to silicate crustal materials on terrestrial planets, which indicates that the lifetime of sediments on Titan’s surface is likely much shorter compared to silicates on Earth due to rapid abrasion into dust.^14^ This means additional mechanisms are needed, such as the combined model that involves periodic abrasion and sintering,^20^ to explain the formation, evolution, and longevity of equatorial dunes on Titan’s surface.

**Table 3:**
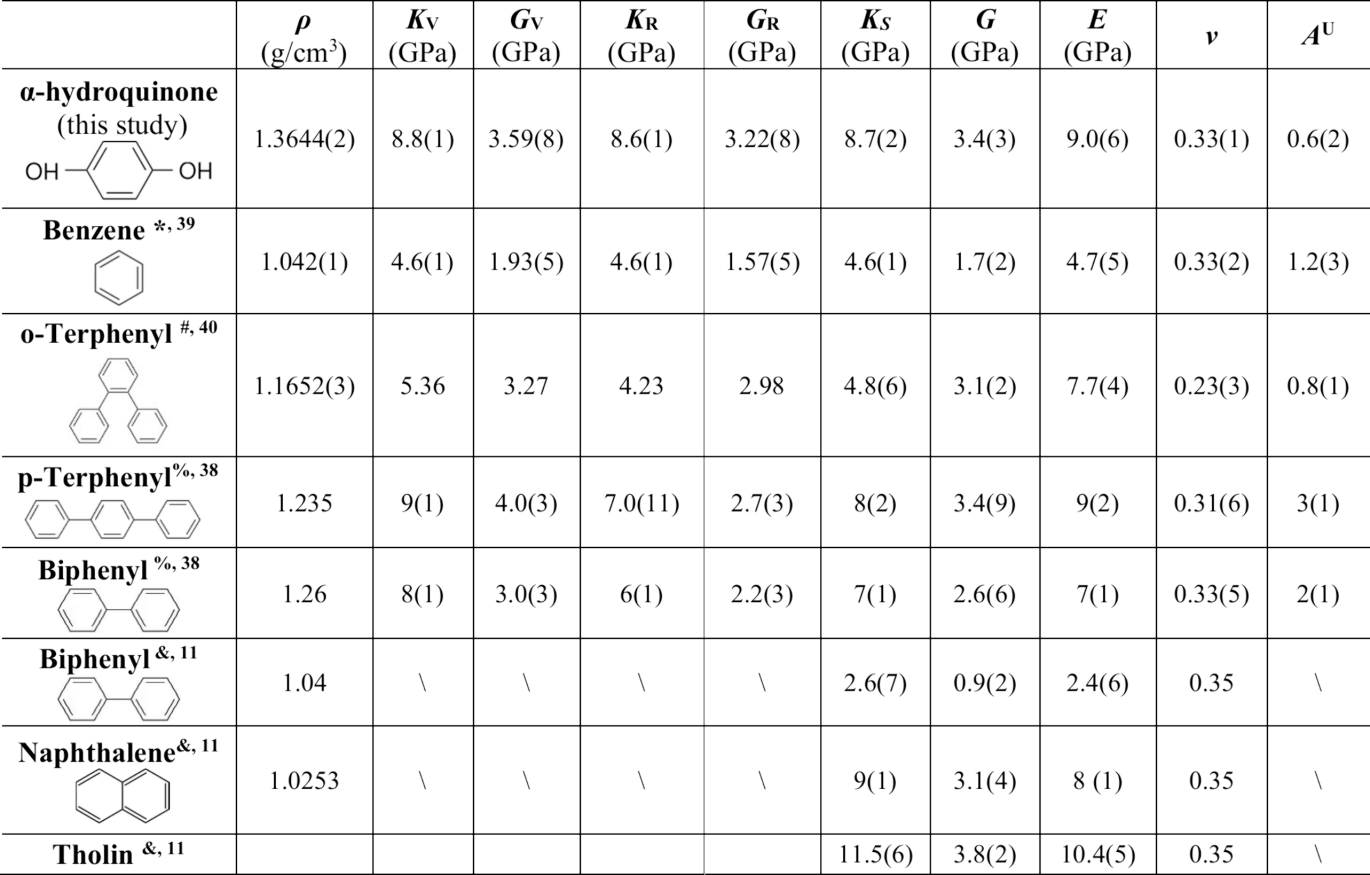
Elastic Properties of Benzene Biphenyl, *p*-Terphenyl, *o*-Terphenyl, Naphthalene, and Tholin Compared to α-Hydroquinone^a^

a Subscripts V and R denote the Voigt and Reuss bounds. *K*_S_ and *G* are calculated as the Voigt–Reuss–Hill average unless otherwise noted. *A*^U^ is calculated on the basis of eq 3. ^*^At 250 K, the density is estimated from a prior study,^44^ uncertainties of *C*_11_, *C*_22_, *C*_33_, *C*_44_, *C*_55_, and *C*_66_ are estimated to be 2%, and uncertainties of *C*_12_, *C*_13_, and *C*_23_ are estimated to be 10%. ^#^The density is estimated from a prior study.^45^ Uncertainties were not given for *C*_*ij*_; thus, the uncertainties of *K*_V_, *G*_V_, *K*_R_, and *G*_R_ were assumed to be 0 when calculating the uncertainties for other quantities. ^%^Uncertainties of *C*_22_, *C*_44_, *C*_66_, and *C*_46_ are estimated to be 3%; uncertainties of *C*_11_, *C*_33_, *C*_55_, *C*_13_, *C*_15_, and *C*_35_ are estimated to be 5%; and uncertainties of *C*_12_, *C*_23_, and *C*_25_ are estimated to be 50%. ^&^*E* was determined using nanoindentation, and *K*_S_ and *G* are calculated on the basis of the reported ν^14^ assuming that the tests were conducted on a homogeneous isotropic polycrystalline aggregate.

## Conclusion

The Dragonfly rotorcraft is expected to arrive at Titan in the mid-2030s and will perform a variety of investigations intended to elucidate Titan’s prebiotic chemistry and habitability. Among these are seismic investigations that will be used to look for quakes caused by tidal deformation of the icy crust. The seismic data will enable modeling to determine the thickness of Titan’s ice shell and interior structure as well as some properties of the near-surface materials.^10^ This modeling can be aided by knowledge of the sound velocities of Titan’s organic material. However, the elastic properties of many Titan-relevant organic materials under Titan conditions are currently unknown. This study serves as a demonstration of the utility of Brillouin spectroscopy in determining wave velocities in organic molecular materials. Future Brillouin spectroscopy studies under cryogenic temperatures can provide vital information for characterizing the mechanical properties of planetary materials in the outer solar system.
